# Mitochondrial K^+^ Transport: Modulation and Functional Consequences

**DOI:** 10.3390/molecules26102935

**Published:** 2021-05-14

**Authors:** Osvaldo Pereira, Alicia J. Kowaltowski

**Affiliations:** Departamento de Bioquímica, Instituto de Química, Universidade de São Paulo, São Paulo 05508-000, SP, Brazil; osvaldo.pereira@usp.br

**Keywords:** mitochondria, potassium, transport, drugs, inhibition, activation

## Abstract

The existence of a K^+^ cycle in mitochondria has been predicted since the development of the chemiosmotic theory and has been shown to be crucial for several cellular phenomena, including regulation of mitochondrial volume and redox state. One of the pathways known to participate in K^+^ cycling is the ATP-sensitive K^+^ channel, MitoK_ATP_. This channel was vastly studied for promoting protection against ischemia reperfusion when pharmacologically activated, although its molecular identity remained unknown for decades. The recent molecular characterization of MitoK_ATP_ has opened new possibilities for modulation of this channel as a mechanism to control cellular processes. Here, we discuss different strategies to control MitoK_ATP_ activity and consider how these could be used as tools to regulate metabolism and cellular events.

## 1. Introduction

Mitochondria are widely, and rightfully, recognized as the hubs of energy metabolism, organelles in which key metabolic pathways (including oxidative phosphorylation, the citric acid cycle, β-oxidation, and part of amino acid metabolism) occur. In mitochondria, nutrient breakdown and oxidation is associated with highly efficient ATP generation through oxidative phosphorylation, which involves the reduction of oxygen to water in the electron transport chain, generation of an inner mitochondrial membrane electrochemical potential due to proton pumping, and ATP production coupled with proton re-entry into mitochondria through the ATP synthase [1].

In the 1960s and 1970s, two further mitochondrial functions were uncovered: the generation of superoxide radical anions and other oxidants derived from it [2,3,4,5], and the uptake and release of Ca^2+^ ions [6,7,8]. Interestingly, the relationship between these two phenomena—redox state and mitochondrial Ca^2+^ transport—was recognized early on [9,10]: oxidized pyridine nucleotides [11] and membrane protein thiols [12] favor Ca^2+^ release from mitochondria.

In the 1990s, mitochondrial function gained attention as a regulator of key cellular survival processes. Mitochondrial intermembrane space proteins were found to be involved in apoptosis when released to the cytosol [13,14]. Furthermore, opening of mitochondrial K^+^ channels was shown to be an effective mechanism to protect against cardiac ischemic damage [15]. These findings and others boosted publications in mitochondrial research, which went from roughly 2000 papers per year to close to 8000 a year today, and still rising [16].

## 2. The K^+^ Cycle

Because intracellular K^+^ concentrations are high, in the range of 140 mM, and the mitochondrial inner membrane has an electrochemical potential in the range of 100 to 200 mV with a negative inside, K^+^ ions are expected to leak through the membrane at biologically relevant rates, despite the high impermeability of this membrane. When K^+^ enters mitochondria in the presence of permeable anions (including phosphate, which is present at millimolar concentrations intracellularly), K^+^ uptake is accompanied by osmotically obligated water and results in organellar swelling [17]. As a result, mechanisms to control K^+^ concentrations in the matrix were predicted to exist when the chemiosmotic theory was developed [18].

Indeed, mitochondria from all tissues tested possess K^+^/H^+^ exchange activity, performed by a protein exchanger that can be pharmacologically modulated [19]. Unfortunately, despite its seminal role in mitochondrial homeostasis, the molecular identity of this exchanger remains debated. Since many key mitochondrial transport pathways were only molecularly identified in the last few years, we hope the molecular identity of this seminal mitochondrial transporter will soon be uncovered.

**Figure 1:**
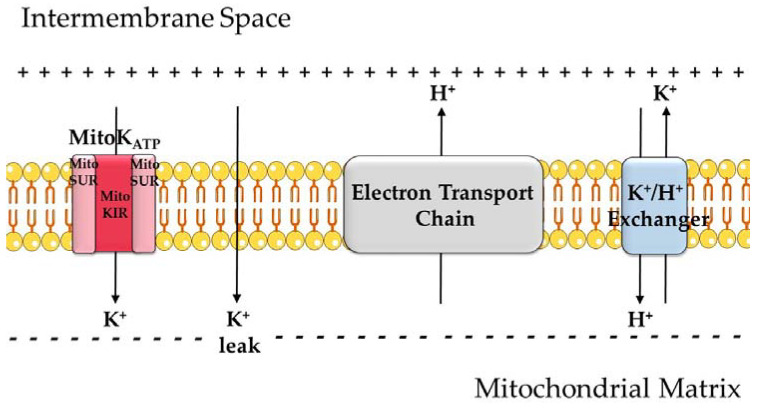
An overview of mitochondrial K^+^ transport. Because of proton pumping by the electron transport chain (ETC) coupled to the reduction of oxygen and oxidation of coenzymes, mitochondria have an electrochemical gradient, with a negatively charged matrix. K^+^ leak across the inner membrane is quantitatively relevant due to the high concentrations of this ion in the cytosol and this electrochemical gradient. K^+^ ions can also enter the matrix through the ATP-sensitive K^+^ channel (K_ATP_ or MitoK_ATP_). The channel is formed by two subunits: MitoKIR (or MitoK) and MitoSUR. K^+^ is removed from the matrix in exchange for H^+^.

Surprisingly, in 1991 Inoue et al. discovered, through patch-clamping experiments, that mitochondria had a regulated K^+^ entry pathway in addition to the K^+^ leak [20]. This pathway was inhibited by ATP in a Mg^2+^-dependent manner [21,22]. The newly described mitochondrial ATP-sensitive K^+^ channel, MitoK_ATP_ (Figure 1), presented many characteristics similar to plasma membrane K_ATP_ channels (CellK_ATP_’s), including inhibition by sulphonylureas [22,23]. Regulation, however, is not identical, as MitoK_ATP_ channels (but not CellK_ATP_’s) are activated by both GTP and GDP, and inhibited by ADP and long-chain acyl-CoA esters [24]. This means MitoK_ATP_’s, unlike CellK_ATP_’s, are not modulated directly by ADP/ATP ratios. Given that the affinity of the channel for ATP is much higher than cellular concentrations and that channel inhibition is also promoted by ADP, MitoK_ATP_ regulation in vivo has been proposed to be controlled by cellular levels of guanine nucleotides and long-chain acyl-CoA [24], as well as by oxidants and antioxidants [25].

Functional properties of MitoK_ATP_ are still being uncovered, but certainly involve volume homeostasis. Because K^+^ leak is dependent on inner membrane potentials and these are variable with cell energy demands, a regulated pathway to ensure K^+^ entry into mitochondria may be necessary to maintain organellar architecture. Volume homeostasis of the mitochondrial matrix through regulated K^+^ uptake is specifically important to maintain the relationship between the inner and outer mitochondrial membranes, since matrix swelling and contraction can affect the interface between these membranes. Matrix volume regulation may also be important for the maintenance of cristae architecture and function [26].

Structurally, early reconstitution studies demonstrated the existence of two subunits necessary to form the holo-MitoK_ATP_. The first is a pore-forming K^+^ channel that was named MitoKIR, and the other is an ATP-binding subunit also known to be a receptor for many pharmacological modulators such as sulphonylureas, therefore called MitoSUR [22,26,27,28]. Excitingly, in late 2019 the molecular identity of these channels was identified [29]. The results are compatible with previous structural studies and led to the identification of the genes coding for both subunits, which were termed MITOK and MITOSUR, respectively. This seminal finding allows us new and more specific genetic approaches toward studying the biology and consequences of these channels, which we hope will begin a new era of mechanistic understanding of mitochondrial K^+^ homeostasis.

Despite the lack of knowledge regarding its molecular identity until recently, MitoK_ATP_ had been extensively explored for its protective properties against ischemia reperfusion in the heart and other tissues using pharmacological tools. Considering the central role that this protein and the K^+^/H^+^ exchanger have in mitochondrial and cellular homeostasis, we believe that, as a result of its recent molecular characterization, this protein could be even further explored as a target for biological modulation and therapeutic interventions. Here, we review the main pharmacological tools employed to modulate the activity of MitoK_ATP,_ to understand its function, signaling responses and therapeutic potential.

## 3. Activating MitoK_ATP_

### 3.1. Pharmacological MitoK_ATP_ Activators

After ATP-sensitive K^+^ transport in mitochondria was described, pharmacological properties of the MitoK_ATP_ were determined through in situ and reconstitution experiments. At that time, cell membrane ATP-dependent K^+^ channels (CellK_ATP_) were also being explored as receptors for modulating molecules, and the properties of both channels were assessed and compared. This led to the finding that CellK_ATP_’s and MitoK_ATP_ presented vastly different affinities for channel activators such as diazoxide, which is not an effective CellK_ATP_ agonist, but opens MitoK_ATP_ at micromolar concentrations [15,27] (see Table 1). Importantly, in order to see pharmacological activation, experiments required the presence of physiological MitoK_ATP_ inhibitors ATP and Mg^2+^. This highlights a logical but often overlooked property of the MitoK_ATP_: it is already active in the absence of physiological ATP or ADP concentrations, so adding agonists under these conditions will not uncover expected intracellular effects of channel agonists.

In the presence of physiological inhibitors, both diazoxide and cromakalim were found to be potent MitoK_ATP_ agonists. Diazoxide is 2000 times more selective toward the mitochondrial channel when compared to plasma membrane counterparts [25]. This selectivity was decisive to identify mitochondrial channels as mediators of cardiac protection against ischemia reperfusion [15]. When used at concentrations that activate MitoK_ATP_, but not CellK_ATP_, diazoxide protects hearts against damage promoted by ischemia followed by reperfusion, a finding that attracted wide attention to the role of mitochondrial K^+^ homeostasis in tissue protection under myriad of stressful conditions [15,30,31,32].

Diazoxide and cromakalim belong to a large group of K^+^ channel opener (KCO) molecules that also includes other compounds such as nicorandil and pinacidil. Although the later activators show some selectivity for MitoK_ATP_ (Table 1), they have mixed effects and can still act on CellK_ATP_’s. Furthermore, KCO effects on CellK_ATP_ vary with cell types. For example, diazoxide can activate CellK_ATP_ in β cells, while pinacidil cannot [33,34]. Moreover, nicorandil also has unspecific vasodilatation effects, since it can be a nitric oxide donor [33]. Diazoxide also displays undesired vasodilatation effects by acting on smooth muscle CellK_ATP_’s [35,36]. As a result, the need to search for even more selective drugs was recognized, and BMS191095 was synthesized based on cromakalim structure as a more specific drug that did not promote vasodilatation and disturb action potentials but still protected murine and canine hearts against ischemic insults [37,38].

Cromakalim and diazoxide act as agonists by interacting with the MitoSUR portion of the channel. This is demonstrated by the fact that isolation of MitoKIR and reconstitution into liposomes generates passive K^+^ transport inhibited by ATP (although much higher concentrations of ATP are needed than with the complete MitoK_ATP_), but none of these KCOs recover ionic flux under these conditions. The results of isolated subunit reconstitution studies thus demonstrate the interaction of the channel agonists with MitoSUR. This same set of experiments also identified *p*-diethylaminoetylbenzoate as an activator of isolated MitoKIR, also efficient in isolated mitochondria [39].

### 3.2. Physiological MitoK_ATP_ Modulation

Although not the central focus of this review, we should stress that in addition to having many well-studied pharmacological activators, MitoK_ATP_ also is physiologically regulated, as these regulators may inspire new pharmacological intervention approaches. Indeed, MitoK_ATP_ channels are controlled by kinases, and are activated in response to PKC agonists [42]. MitoK_ATP_ channels are also modulated by respiratory complex II activity, and may respond to endogenous complex II inhibitors such as malonate [43].

MitoK_ATP_ channel activity is strongly sensitive to redox state. While oxidants such as superoxide radicals, H_2_O_2_ and *S*-nitrosothiols activate the channels, thiol reductants such as *N*-acetylcysteine, 2-mercaptopropionylglycine and dithiothreitol [25] inhibit the channel directly, suggesting it has redox-sensitive thiols. NADPH is also a MitoK_ATP_ inhibitor [44,45]. This redox-regulation of the channel is in keeping with its role in modulating mitochondrial oxidant production [46,47]. We hope that the now uncovered molecular identity of the channel will help future studies in determining the mechanisms of MitoK_ATP_ modulation by these compounds, including the identification of its redox sensors. These studies may also open new windows of opportunities for the design of novel KCOs.

### 3.3. Physiological Consequences of MitoK_ATP_ Opening

**Figure 2:**
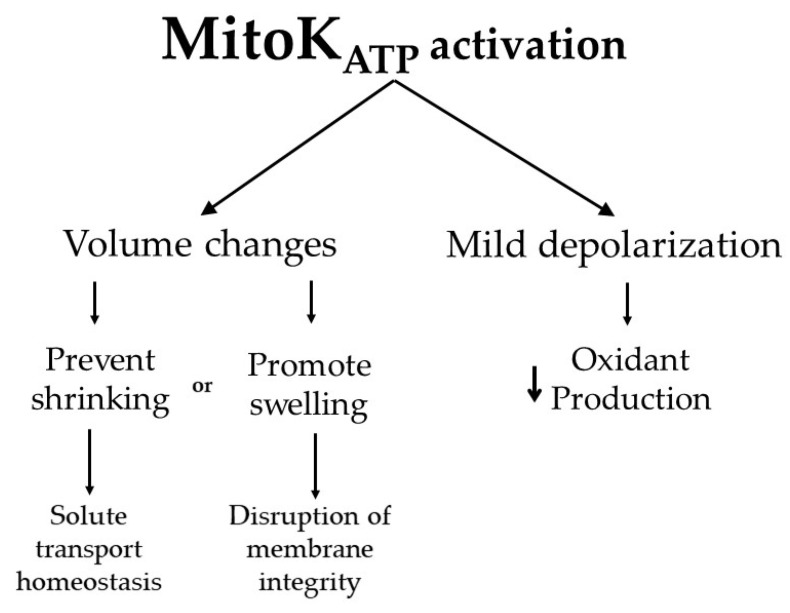
Functional consequences of MitoK_ATP_ activation. K^+^ entry in the mitochondrial matrix through MitoK_ATP_ leads to the uptake of water, which changes mitochondrial volume. This can be important to maintain proper membrane transport properties, while in extreme conditions, excessive swelling (often not specifically a consequence of MitoK_ATP_ activity) can lead to membrane integrity disruption. Dilution of matrix components by water uptake also leads to the activation of K^+^ exit in exchange for protons, which leads to proton exit as the net product, mildly uncoupling mitochondria. This uncoupling prevents oxidant production by the electron transport chain.

As discussed previously, activating MitoK_ATP_ allows K^+^ entry into the mitochondrial matrix which, accompanied by anion transport, promotes mitochondrial swelling though uptake of osmotically obligated water. This is important for the maintenance of mitochondrial structure and regulation of the intermembrane space architecture [48]. Thus, activating the MitoK_ATP_ can prevent structural damage to mitochondria caused by excessive contraction under pathological situations and preserve transport properties through the mitochondrial membrane, including the transport of ADP and ATP (Figure 2) [44,45].

K^+^ cycling in mitochondria is expected to result in a decrease in inner membrane potentials through the combined action of K^+^ entering through MitoK_ATP_ and exiting in exchange for protons, uncoupling electron transport from ATP synthesis. Uncoupling promoted by this cycling is limited due to low K^+^ transport rates through MitoK_ATP_ in most tissues, and it promotes only a 1–2% decrease in mitochondrial inner membrane potentials in the heart [49]. Despite the small changes in ΔΨ_m_, even low levels of uncoupling can significantly modify oxidant production rates (Figure 2) [49,50].

Mitochondria produce oxidants through a few distinct pathways, and a very important source of reactive oxygen species is electron leakage in the electron transport chain, which can generate superoxide radical anions and hydrogen peroxide [51]. High ΔΨ_m_ is needed for electron leakage, as it often depends on reverse activity of mitochondrial complexes [5,52,53], which is thermodynamically feasible only at high ΔΨ_m_. Indeed, MitoK_ATP_ activation was found to significantly decrease mitochondrial H_2_O_2_ release in different tissues [45]. Further studies indicated that, in addition to regulating mitochondrial oxidant production, MitoK_ATP_ channels were also redox-sensitive, opened by superoxide radicals, H_2_O_2_ and *S*-nitrosothiols, and closed by thiol reductants and NADPH [25,44]. As a result, they participate in an elegant redox-sensitive pathway regulating mitochondrial oxidant production. This, added to the volume modulating functions of MitoK_ATP_, is probably the most important physiological role for this channel (Figure 2).

While MitoK_ATP_ opening effects are sufficient to significantly impact mitochondrial oxidant production, the small changes in ΔΨm are insufficient to change isolated mitochondrial Ca^2+^ uptake under physiological respiring conditions. However, Ca^2+^ uptake is decreased by MitoK_ATP_ activity in non-respiring mitochondria in which ΔΨm is supported by the reverse activity of the ATP synthase [52,53]. Excessive Ca^2+^ entry can lead to accumulation of the ion in the mitochondrial matrix and opening of the mitochondrial permeability transition pore (MPTP). This causes a disruption of mitochondrial inner membrane integrity that promotes cell death [54,55]. Since MPTP opening is facilitated by oxidation of protein thiols [12] and enhanced mitochondrial oxidant production [11], it is not surprising that there is evidence to support that MitoK_ATP_ activation can prevent MPTP opening [56].

### 3.4. Beneficial Effects of MitoK_ATP_ Activators under Pathological Conditions

MPTP opening leads to loss of oxidative phosphorylation capacity as well as release of pro-death mitochondrial proteins, and is a cause of ischemic tissue damage [57]. MitoK_ATP_’s protective effects against MPTP are certainly part of the reason why KCOs are strongly protective against ischemic damage in the heart and other tissues [15,31].

During ischemia, the interruption of proper blood flow leads to a decrease in oxygen supply that suppresses the activity of the electron transport chain; consequently, proton motive force is quickly dissipated. Under these conditions, ATP synthase can work its reverse activity and hydrolyze ATP, an activity that partially re-establishes ΔΨ_m_ [58,59]. As cytosolic Ca^2+^ concentration rises during ischemia [60], this increase in the driving force (ΔΨm) can promote mitochondrial Ca^2+^ accumulation and lead to MPTP opening. Upon reperfusion, cardiac cells are known to present an increase in oxidant production induced by ischemia [61], which also favors MPTP opening.

Activation of MitoK_ATP_ has been proposed to block ischemic damage by maintaining proper membrane transport properties and preventing ATP entry to the mitochondrial matrix [44]. This blocks ATP hydrolysis by the reverse activity of the ATP synthase, with transient re-establishment of ΔΨ_m_ that could prevent ATP loss and excessive mitochondrial Ca^2+^ accumulation [62]. Moreover, pharmacological activators of MitoK_ATP_ can also block excessive production of oxidants in mitochondria, preventing myocadiac ischemic damage. The highly specific MitoK_ATP_ activator BMS-191095 was also shown to additionally act to prevent ischemic damage beyond cardiac cell effects by inhibiting platelet aggregation [32].

Ischemic protection by MitoK_ATP_ opening occurs not only in the heart: results in murine neurons and brain [63] show that diazoxide can protect these cells against hypoxic damage associated with ischemia by preventing repolarization of mitochondria [31].

MitoK_ATP_-mediated protection is also not limited to ischemia reperfusion. Indeed, diazoxide treatment also protected murine heart against hypertrophy by controlling oxidant generation [64], and prevented neuronal injury in models of the metabolic disease methylmalonic acidemia by preventing MPTP opening [65,66]. Overall, given its effects modulating mitochondrial redox state and preventing MPTP without overt changes in oxidative phosphorylation, MitoK_ATP_ can act as an effective protective pathway under many different pathological conditions.

Indeed, protective mechanisms mediated by MitoK_ATP_ are also part of endogenous signaling pathways. Preconditioning is a protective intervention where hearts undergo brief and non-damaging periods of ischemia [45], which significantly decrease damage associated with a subsequent larger and typically damaging ischemic event. The protection promoted by preconditioning can be blocked by MitoK_ATP_ inhibitors [33,34,67,68], indicating that this protein actively participates in this process. Another key protein in preconditioning signaling is protein kinase C (PKC). PKC activation leads to an increase in MitoK_ATP_ activity, and PMA, an activator of PKC, has been shown to indirectly promote opening of MitoK_ATP_ in intact cells [42].

## 4. Inhibiting MitoK_ATP_

Prior to the identification of MitoK_ATP_ as their receptor, molecules such as glyburide (also known as glybenclamide) and 5-hydroxydecanoate (5-HD) were known to reverse ischemic protection induced by KCOs or preconditioning [67,68]. Interestingly, after MitoK_ATP_ was identified, initial studies in isolated mitochondria failed to see selective inhibition of MitoK_ATP_ by these compounds [69]. Later studies showed that, counterintuitively, MitoK_ATP_ could not be inhibited pharmacologically when open by the absence of physiological inhibitors. Instead, channel modulation by glyburide and 5-HD required incubation in the presence of physiological inhibitors such as ATP and Mg^2+^ or long chain acyl-CoA, in addition to the presence of either physiological activators, such as guanine nucleotides, or pharmacological agonists, such as diazoxide [70]. Under these appropriate conditions, both 5-HD and glyburide were shown to effectively close the channel.

Because of its selectivity, 5-HD became the most employed tool as a MitoK_ATP_ antagonist. Both molecules are proposed to act through the MitoSUR subunit and cannot inhibit MitoKIR alone [71]. This is expected for glyburide, considering it is a sulphonylurea [71]. 4-aminopyridine, a general K^+^ inhibitor, requires much higher concentrations than all other inhibitors listed in order to function on MitoK_ATP_ [20]. Another well-described modulator known to act directly on the MitoKIR subunit is tetraphenylphosphonium (TPP^+^) [49], although this is not a selective inhibitor, and has been shown to act in CellK_ATP_’s as well [72]. This is of interest, since TPP^+^ is often used to monitor mitochondrial inner membrane potentials with electrodes [73]. Information on MitoK_ATP_ inhibitors mentioned here are listed in Table 2.

## 5. Modulation of the Mitochondrial K^+^/H^+^ Exchanger

The activity of a mitochondrial K^+^/H^+^ exchanger has been predicted since the proposal of the chemiosmotic theory [18]. An exit route is necessary for K^+^, since it would be predicted to leak across the inner membrane in the presence of the electrochemical gradient. Experimentally, K^+^/H^+^ exchange was initially observed as K^+^ efflux from mitochondria caused by suspending these organelles in hypotonic sucrose [74]. This already indicates the importance of this pathway in maintaining mitochondrial structure and counterbalancing osmotic swelling caused by K^+^ uptake.

Physiologically, K^+^/H^+^ exchange activity is known to be reversibly inhibited by intramitochondrial Mg^2+^ [75]. When K^+^ enters the matrix through leakage or MitoK_ATP_, it is accompanied by osmotically obligated water, promoting swelling. This dilutes intramitochondrial Mg^2+^ and activates K^+^/H^+^ exchange, ensuring volume homeostasis [75].

Some tools have been proposed in the literature to control exchanger activity. Quinine and quinacrine are reversible inhibitors at relatively low concentrations [76], while *N*,*N*’-dicyclohexylcarbodiimide (DCCD) can irreversibly inactivate the exchanger [77]. DCCD is a non-selective drug that reacts with carboxylic groups in several bioenergetically relevant targets, including other ion transport proteins [78], while quinine and quinacrine are K^+^ transport modulators, but do not have strict selectivity for mitochondrial K^+^/H^+^ exchange. Interestingly, DCCD only reacts with the active mitochondrial K^+^/H^+^ exchanger; therefore Mg^2+^ and quinine protect the exchanger from DCCD inactivation [79].

Mitochondrial K^+^/H^+^ exchange inhibitors block excessive mitochondrial contraction in isolated mitochondria [76]. Although this suggests an interesting role of the exchanger in maintaining mitochondrial architecture and preserving inner membrane transport properties, its potential as a target for therapeutical interventions has not yet been explored, mostly due to the lack of specific pharmacological tools to modulate this transport and the lack of knowledge regarding the molecular identity of the exchanger.

## 6. Conclusions

Considering the central relevance that mitochondria have in nearly every cell in the human body, the mitochondrial K^+^ cycle, and more specifically MitoK_ATP_ as a key component of it, are powerful targets to control key mitochondrial functions, including the preservation of volume, architecture and redox state. Given the already numerous known MitoK_ATP_ pharmacological regulators and the potential for new drug discovery and design, as well as the recent molecular identification of the channel protein subunits, we believe the applications for mitochondrial KCOs will expand largely in the near future.

## Figures and Tables

**Table 1:** MitoK_ATP_ activators.

Compound Name	Effective Concentrations (μM)	References
Diazoxide	30	Garlid et al., 1997 [15]
Pinacidil	100	Crestanello et al., 2000 [40]
Nicorandil	100	Teshima et al., 2003 [41]
Cromakalim	30	Garlid et al., 1997 [15]
BMS191095	10	Grover et al., 2001 [38]
*p*-diethylaminoetylbenzoate	100	Mironova et al., 2004 [39]
phorbol 12-myristate 13-acetate (PMA)	0.2	Sato et al., 1998 [42]

**Table 2:** MitoK_ATP_ inhibitors.

Compound Name	Concentrations Employed (μM)	Reference
5-Hydroxydecanoate	45–75	Jaburek et al., 1998 [70]
Glyburide	5–6	Inoue et al., 1991 [20], Jaburek et al., 1998 [70]
Tetraphenylphosphonium	0.1	Mironova et al., 2004 [40]
4-aminopyridine	5 × 10^3^	Inoue et al., 1991 [20]

## Data Availability

Not applicable.
